# Complete mitochondrial genome sequence of the genus *Austinograea* (Malacostraca: Brachyura: Bythograeidae) and its phylogenetic analysis

**DOI:** 10.1080/23802359.2020.1821823

**Published:** 2020-10-05

**Authors:** Peng Xu, Yadong Zhou, Chunsheng Wang

**Affiliations:** a Key Laboratory of Marine Ecosystem Dynamics, Second Institute of Oceanography, Ministry of Natural Resources, Hangzhou, PR China; b School of Oceanography, Shanghai Jiao Tong University, Shanghai, PR China; c State Key Laboratory of Satellite Ocean Environment Dynamics, Hangzhou, PR China

**Keywords:** Indian Ocean, mitogenome, phylogeny, Eubrachyura

## Abstract

*Austinograea* species are restricted to hydrothermal vents and are typically considered to be omnivorous predators in vent communities. Here we present the complete mitochondrial genome of *Austinograea* sp. which was collected from Carlsberg Ridge, the mid-ocean ridge in the northwestern Indian Ocean. The genome is 15,584 bp in length with a 68.11% AT content. It contains 13 protein-coding genes (PCGs), 2 ribosomal RNA genes, and 22 transfer RNA genes. Phylogenetic analysis shows that the present species is closest to *Austinograea rodriguezensis*. This study contributes to further phylogenetic analysis within Eubrachyura.

All identified species in the genus *Austinograea* except *Austinograea rodriguezensis* colonize hydrothermal vents in the western Pacific Ocean at present and the factors that drove the adaptation and speciation of *Austinograea* remain unknown (Hessler and Martin 1989; Guinot 1990; Tsuchida and Hashimoto 2002; Mateos et al. 2012; Kim et al. 2013; Guinot and Segonzac 2018). The examination of phylogenetic relationships among *Austinograea* species could help to clarify the interaction between biological evolution and geographical processes at deep-sea hydrothermal vents (Lee et al. 2019). In this study, the complete mitochondrial genome from a deep-sea *Austinograea* sp. was characterized, and its relationship with closely related species was investigated.

The specimen was collected from Carlsberg Ridge, the mid-ocean ridge in the northwestern Indian Ocean (60°31′48″E, 6°21′36″N, 2920 m depth) using the Chinese manned submersible *Jiaolong*, and was identified as *Austinograea* sp. based on its morphological characters. The specimen (SRSIO17030301) and its DNA (DNASIO17030301) are deposited in the Sample Repository of the Second Institute of Oceanography, Ministry of Natural Resources, Hangzhou, China. DNA was extracted with QIAamp Tissue Kit (QIAGEN, Hilden, Germany) and mitochondrial DNA was amplified with a DNA REPLI-g Mitochondrial DNA Kit (QIAGEN, Hilden, Germany) as directed by the manufacturer. Library construction and sequencing were performed by Biozeron (Biozeron, Shanghai, China) using the Illumina Hiseq4000 sequencing platform (Illumina, San Diego, CA).

The complete mitogenome sequence of *Austinograea* sp. is 15,584 bp in length with a 68.11% AT content. It contains 13 protein-coding genes, 2 ribosomal RNA genes, and 22 transfer RNA genes. Among the 37 genes, both rRNA genes (*rrnL* and *rrnS*) are encoded on the light strand, as in the other crustacean mitochondrial genomes. Eight tRNA genes (*trnC-tgc*, *trnF-ttc*, *trnH-cac*, *trnL1-cta*, *trnP-cca*, *trnQ-caa*, *trnV-gta*, and *trnY-tac*) are encoded on the light strand. Only four protein-coding genes (PCGs) (*nad1*, *nad4*, *nad4L*, and *nad5*) are encoded on the light strand, whereas the other nine PCGs are located on the heavy strand. Eight PCGs (*cox1*, *cox2*, *atp8*, *atp6*, *cox3*, *nad4*, *cob*, and *nad2*) are initiated by ATG. Two PCGs (*nad4L* and *nad6*) are started by ATT. The other three PCGs (*nad1*, *nad3*, and *nad5*) are initiated by ATA, ATC, TTG, respectively. Ten PCGs (*nad3*, *cox1*, *cox2*, *atp6*, *cox3*, *nad4*, *cob*, *nad2*, *nad4L*, and *nad6*) terminate with the typical TAA as stop codon, while the remaining three PCGs (*nad1*, *atp8*, and *nad5*) end with TAG. A total of 22 transfer RNA genes range in size from 62 to 73 bp. The mitogenome of *Austinograea* sp. has been deposited in GenBank under accession number MT855983.

To reveal the phylogenetic relationship of *Austinograea* sp. with the other crabs, a total of 22 species with 23 mitochondrial genomes from Eubrachyura were obtained from the GenBank database. The mitochondrial PCGs were used for phylogenetic analysis by maximum likelihood (ML) method. In the phylogenetic tree, *Austinograea* sp. was closely related to *A. rodriguezensis*, and they clustered with *A. alayseae* into a branch. Meanwhile, *Austinograea* had a close relationship with *Gandalfus* in Bythograeoidea (Figure 1). In this study, we present the complete mitochondrial genome sequence of *Austinograea* sp., which would contribute to further phylogenetic and comparative mitogenome studies of Eubrachyura. Furthermore, more mitochondrial genomic data of undetermined taxa and further analysis are required to reveal phylogeny and evolution of crabs.

**Figure 1.**
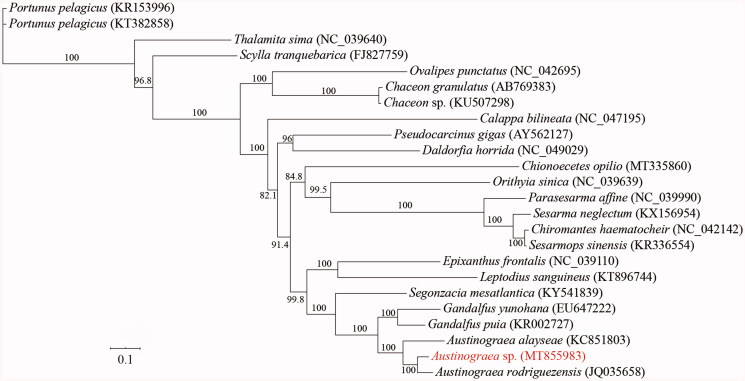
Phylogenetic tree of *Austinograea* sp. and other mitochondrial genomes from Eubrachyura based on mitochondrial PCGs.

## Data Availability

All sequences generated or used in the present study are deposited in NCBI GenBank (www.ncbi.nlm.nih.gov/) and the accession numbers are detailed in Figure 1.
